# Observational study of laryngoscopy plus flow‐volume loops during exercise

**DOI:** 10.1002/ccr3.1375

**Published:** 2018-03-04

**Authors:** Paolo T. Pianosi, Diana M. Orbelo, Shelagh A. Cofer

**Affiliations:** ^1^ Department of Pediatric & Adolescent Medicine Mayo Clinic 200 First St. SW Rochester Minnesota 55905; ^2^ Department of Otorhinolaryngology Mayo Clinic 200 First St. SW Rochester Minnesota 55905

**Keywords:** Dyspnea, exercise, flow‐volume loop, laryngoscopy, stridor, vocal cord dysfunction

## Abstract

Laryngoscopy is the gold standard to diagnose exercise‐induced laryngeal obstruction, though inspiratory flow‐volume loop may provide a clue. We combined tidal flow‐volume loop analysis *plus* laryngoscopy during exercise and found that cigar‐shaped – *not* flattened – inspiratory loops are associated with obstruction. Pursed‐lip breathing slows inhalation thereby reducing vocal fold adduction.

## Introduction

The advent of continuous laryngoscopy during exercise (CLE) 1 permitted identification of exercise‐induced laryngeal obstruction (EILO) 2. The clinical problem is now more broadly recognized to involve other glottic structures, giving rise to this new nomenclature. Although CLE has become the diagnostic gold standard, earlier literature suggested that flat or truncated inspiratory flow‐volume loop is abnormal at rest 3, 4, during 5, or after 6, 7 an episode; although this has recently been questioned 8. Management typically consists of instructions on specific breathing maneuvers from a trained speech‐language pathologist, but a systematic review of this approach concluded that there is pressing need for experimental studies to further the evidence base 9. Pediatric patients evaluated at Mayo Clinic for work‐up for exertional dyspnea undergo a maximal exercise test with CLE combined with tidal flow‐volume loop analysis. This coupling of visualizing the larynx plus tidal flow‐volume loops, and hearing accompanying stridor, revealed unique clinical‐pathologic correlations that elucidate pathophysiology and treatment principles of EILO.

## Methods

### Test procedures

Patients reported to the exercise laboratory with instructions to either fast for 2 h prior to the test. Subjects performed spirometry immediately prior to exercise on the same MedGraphics system used for the exercise test while seated on the cycle ergometer with the best effort so defined chosen as the maximum expiratory flow‐volume envelope for exercise. Subjects next performed a maximal cardiopulmonary exercise test on an electrically braked, cycle ergometer. Patients were encouraged to exercise to volitional exhaustion to achieve criteria implying maximal effort. Heart rate and SpO2 were monitored continuously with 12‐lead ECG and pulse oximetry, respectively.

### Ventilatory measurements during exercise

Ventilation and gas exchange were measured breath‐by‐breath via mouthpiece using MedGraphics CPX/D (Breeze Software, Medical Graphics Corp, St. Paul, MN) that employs a Pitot tube to measure flow, electronically integrated to give minute volume. The system corrects for drift that occurs when there are differences between inhaled and exhaled volumes. The Breeze^©^ manual states that the program measures exercise tidal flow‐volume loop according to method described by Johnson et al. 10 at Mayo Clinic. In short, the degree of expiratory flow limitation was obtained by aligning a tidal breath during exercise within the maximum flow‐volume envelope obtained at rest prior to the test. Alignment was accomplished by having subjects perform an inspiratory capacity maneuver from resting end‐expiratory lung volume, assuming no change in total lung capacity with exercise. Inspiratory capacity maneuvers were performed at rest, during a 3‐min warm‐up at the initial workload of the protocol, then every other workload. Resting maneuvers were rehearsed until the subject appeared comfortable with, and capable of, performing them satisfactorily. The principal variable one can obtain pertinent to this report is inspiratory flow and inspiratory flow reserve (Figure 2 in reference 11).

### CLE

A fiber optic endoscope was inserted through an anesthetized nostril, advanced through the velopharyngeal port until the larynx was in full view. Once scope position was confirmed and tolerated by the subject, it was taped to the nostril and then suspended via an adjustable clamp system. The scope was repositioned as needed to achieve good visualization of the larynx throughout the exercise test and was left in place into the cool down phase to allow for any additional laryngeal obstruction that might occur after the subject came off the mouthpiece. Nose clips occluded the contralateral nostril.

## Clinical Vignettes

### Patient 1

**Figure 1:**
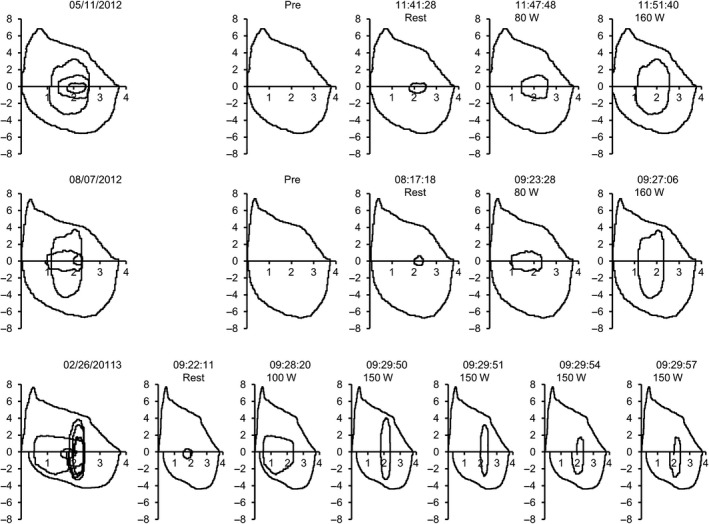
Composite flow‐volume loops of three serial exercise tests in patient 1 proceeding from top to bottom, initial to final tests. Note change in shape of flow‐volume loops from broader ellipse of initial test to cigar‐shaped shaped loop at peak exercise of final test.

A 15‐year‐old girl with 4 months of dyspnea while playing soccer was referred for evaluation. Symptoms started with tightness across the lower costal margin followed by a sense of throat constriction accompanied by noisy inhalation. Spirometry and chest radiograph were normal. She did not respond to initial speech‐language therapy treatment that involved coaching to slow inhalation by purse‐lipped inspiration and attempts to reduce upper body tension by instructing patient to relax her shoulders and engage diaphragmatic breathing. Exercise bronchoprovocation and methacholine challenge were negative, and exhaled nitric oxide was normal. She underwent an incremental exercise test to voluntary exhaustion on a cycle ergometer with CLE. Results of her initial exercise test showed significant inward rotation of the arytenoids as they prolapsed. Vocal folds remained open, but as she continued incremental exercise, arytenoid prolapse increased and was accompanied by inspiratory stridor. Based on this, she underwent right aryepiglottic fold division plus corniculate cartilage and mucosal amputation, as this is often sufficient to open the airway enough to relieve obstruction. The patient continued to experience exertional dyspnea and stridor, prompting a second test 5 months later. Peak work and heart rate were similar, but peak minute volume was higher, and end‐tidal PCO_2_ was lower (Table 1). CLE showed less flipping of the remaining arytenoid tissue, but she again developed mild stridor toward the end of the test. She therefore underwent the same surgical intervention on the left side. Despite bilateral medial arytenoidectomy, she continued to have episodic exertional dyspnea and stridor refractory to behavioral techniques of pursed‐lip and abdominal breathing. A third maximal exercise test with CLE was therefore performed. During light to moderate exercise, she manifested appropriate breathing strategy with good thoracoabdominal movement. There was only mild tipping of the remaining arytenoids, which did not compromise her airway. However, at maximal exercise, she suddenly began to pant and demonstrated paradoxical vocal fold movement with accompanying stridor over the next minute. This unexpected development was associated with an abrupt change in her tidal exercise flow‐volume loops (Fig. 1).

**Table 1:** Peak exercise values for minute volume (*V*_E_, L/min), breath rate (BR, breaths/min), and end‐tidal CO_2_ tension (PetCO_2_, mmHg)

Test	*V*_E_	BR	PetCO_2_
1	83	58	32.6
2	106	81	27.1
3	120	>100	24.0

### Patient 2

**Figure 2:**
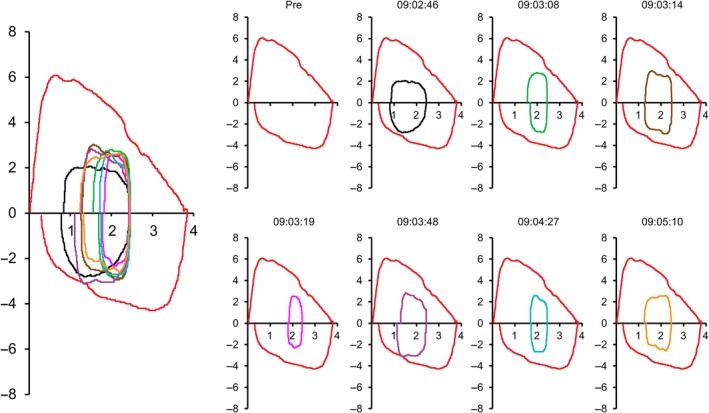
Selected flow‐volume loops with time stamps obtained in patient 2. Note how this individual alternated between broader elliptical vs. cigar‐shaped shaped flow‐volume loops over <2 min of incremental exercise.

**Figure 3:**
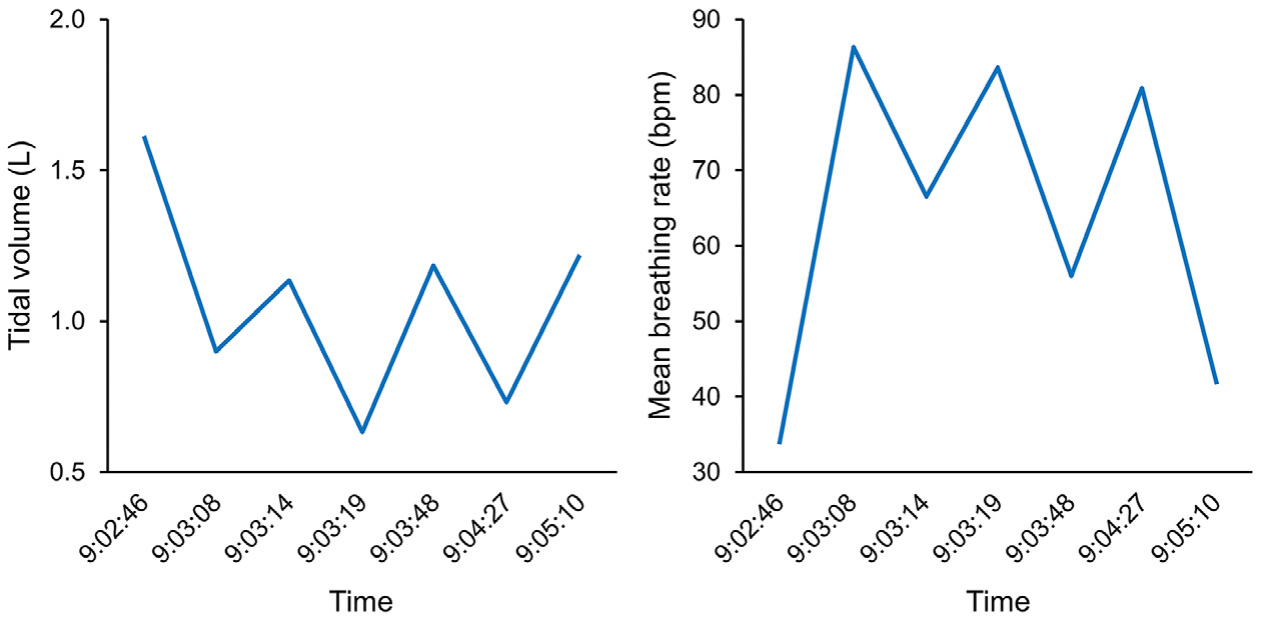
Plot of reciprocal relationship between tidal volume and respiratory rate (5‐breath means spanning time stamp) illustrating the rapid, shallow breathing pattern but maintaining minute volume, aligned on *x‐*axis with time of flow‐volume loops in Figure 2.

A 13‐year‐old girl was evaluated for 9‐month history of exertional dyspnea. She reported difficulty inhaling accompanied by stridor during running drills culminated by presyncopal symptoms that improved with several minutes of rest. There was no history of chronic cough or wheeze, but her methacholine challenge was positive, triggering a 25% fall in FEV_1_ accompanied by throat tightening, reportedly that which she experienced with vigorous exertion. She underwent a maximal exercise test challenge with CLE that demonstrated intermittent stridor during heavy exercise accompanied by paradoxical vocal fold movement (Videos S1 and S2) coincident with an abrupt change in her tidal exercise flow‐volume loops (Fig. 2). Post‐test analysis showed breath rate rose and fell, reaching its zenith coincident with stridor, followed by resolution of stridor as respiratory rate troughed as abruptly as it peaked (Fig. 3).

## Discussion

A recent review on EILO concluded that there remains need for more research elucidating the underlying pathogenesis and optimum management 12, 13. Morris and Christopher 14 laid down three criteria to establish a diagnosis: (1) noisy breathing and dyspnea; (2) laryngoscopic evidence of inspiratory vocal cord adduction; and (3) confirmatory pulmonary function test findings of an abnormal inspiratory flow‐volume loop or absence of airway hyperresponsiveness. However, these criteria can be questioned because EILO often coexists with asthma 15, 16 or stridor can occur without conditions 2 and 3 above 17. Our two patients illustrate this diversity and highlight the complementary role of CLE coupled with tidal flow‐volume loop analysis during exercise. The concept of EILO is continually evolving as our understanding increases with more widespread use of CLE during exercise 18. Flow‐volume loops obtained at rest may provide a clue in perhaps 25% of patients 15, but this is a nonspecific finding, and their utility during exercise has not been demonstrated 13.

Exercise‐induced laryngeal obstruction is more common in females 15, 19 likely because the female larynx is shorter and narrower such that the glottic opening is smaller than in males 20. Therefore, at similar flow rates, the female must develop higher velocities as air penetrates the glottic aperture. Flow through the trachea is laminar at low tidal flow rates, which maintains a distending pressure on the airway wall, but becomes turbulent once critical velocity is exceeded, particularly as air passes the vocal folds producing the so‐called laryngeal jet 21. The shape of the glottic aperture (circular, elliptical, and triangular) influences patterns of turbulent flow within the glottis and the location of the laryngeal jet 22. Turbulent flow results in focal eddies (especially near the cords) resulting in loss of distending pressure. EILO is most severe at peak exercise 23 when minute volume reaches its zenith. That said, flow rates explain only part of pathogenesis of EILO induction – glottic compliance and vocal fold abduction (in the case of paradoxical vocal fold movement) also play a role. Patients with unilateral vocal fold paralysis who underwent Teflon injection of the affected vocal cord had significant improvement in inspiratory airflow afterward, implying that increased stiffness of the vocal fold imparted by Teflon reduced its tendency to prolapse into the glottic airstream 24.

The sequence of events in our patients implies breathing pattern is critical to developing stridor. As breathing becomes more shallow but rapid, borne out by tidal flow‐volume loop, EILO consequently develops. EILO can occur either due to prolapse of supraglottic structure if these tissues are not sufficiently stiff (i.e., noncompliant); or if laryngeal abductors cannot adequately compensate for collapsing forces induced by turbulent flow across the cords. Patient 1 had no signs of laryngeal inflammation on repeated examinations nor did she have asthma, providing no clues regarding potential precipitants or comorbidities 13. We surmise that she exhibited a learned behavior over months which adversely affected her breathing pattern: rapid, shallow breaths preceded and likely triggered EILO. This rapid, shallow breathing pattern as a catalyst was verified in patient 2, who flipped in and out of paradoxical vocal fold movement dependent on breathing pattern. Patient 1 may have experienced a panic reaction at peak exercise in her third test, but patient 2 demonstrates panic is not a prerequisite for adopting this breathing pattern. Note that inspiratory limbs of each patients' flow‐volume loop were not flattened or truncated. The normal flow‐volume loop during exercise is best described as rectangular or elliptical. As this configuration changes to more cigar‐shaped, that is, with its major axis (flow) dimension greatly exceeding its minor axis (tidal volume) dimension, fluid dynamic forces cause paradoxical vocal fold movement. It follows that slowing inspiratory flow rate, such as occurs with pursed‐lip breathing can restore an elliptical flow‐volume profile and reduce forces causing vocal fold adduction.

## Conflict of Interest

None declared.

## Authorship

PP: supervised exercise tests and wrote manuscript. DO: performed CLE and prepared videos. SC: performed surgical interventions on patient 1.

## Supporting information


**Video S1**. This clip was abstracted from third exercise test recording of patient 1 and shows paradoxical vocal fold movement with accompanying audio demonstrates inspiratory stridor.

**Video S2**. This shows actual glottis configuration changes over similar time frame (~150 sec) as flow‐volume loops shown in Figure 2 were obtained. This patient's glottis alternates between normal, wide‐open configuration, and paradoxical vocal fold movement. Unfortunately, audio malfunction precludes demonstration of simultaneous inspiratory stridor.
